# Drug–Drug interactions of docetaxel in patients with breast cancer based on insurance claims data

**DOI:** 10.1371/journal.pone.0287382

**Published:** 2023-06-16

**Authors:** Kwang-Hee Shin, Young-Mi Ah, Sang Hun Cha, Hye Duck Choi

**Affiliations:** 1 College of Pharmacy, Research Institute of Pharmaceutical Sciences, Kyungpook National University, Daegu, Republic of Korea; 2 College of Pharmacy, Yeungnam University, Gyeongsan, Gyeongbuk, Republic of Korea; 3 Department of Statistics, College of Natural Sciences, Kyungpook National University, Daegu, Republic of Korea; Kyung Hee University School of Medicine, REPUBLIC OF KOREA

## Abstract

Despite an increase in the use of targeted anticancer drugs and immunotherapy, cytotoxic anticancer drugs such as docetaxel continue to play a clinically important role. The aim of this study was to evaluate drug–drug interactions between docetaxel and coadministered medicines in patients with breast cancer a claims database. The Health Insurance Review and Assessment Service (HIRA) database (2017 to 2019) was used in this study. We evaluated the risk of neutropenia (defined using receipt of granulocyte colony-stimulating factor (G-CSF) prescriptions) under docetaxel administration or the coadministration of docetaxel and an interacting anticancer drug (predefined based on approval information obtained from the Korean Ministry of Food and Drug Safety and the Lexicomp electronic database). The propensity score matching method was applied to balance covariates in the case (patients with G-CSF prescriptions) and control (patients without G-CSF prescriptions) groups. We identified 947 female patients with breast cancer prescribed with docetaxel and excluded 321 patients based on inclusion criteria. Of the remaining 626 patients, 280 were assigned to the case group and 346 to the control group. Predefined drugs were coadministered to 71 (11.3%) patients during the 7-day period before and after the administration of docetaxel. Adjusted odds ratios (ORs) calculated using the logistic regression model applied to the propensity score matching showed no significant difference between the administration of docetaxel alone and docetaxel coadministration (adjusted OR, 2.010; 95% confidence interval, 0.906, 4.459). In conclusion, we suggest that coadministration of docetaxel and a predefined interacting drug are not associated with G-CSF prescription.

## Introduction

Docetaxel is a conventional chemotherapeutic agent recommended for the treatment of various cancer diseases, such as breast cancer, nonsmall-cell lung cancer, ovarian cancer, and gastric cancer [1–5]. Despite the increasing interest in new anticancer drugs such as targeted agents and immunotherapy, cytotoxic anticancer drugs like docetaxel are still used in clinical settings [2, 3]. For instance, combination regimens, which includes docetaxel, are recommended as first-line therapies for patients with locally advanced or metastatic breast cancer [2].

Since the drug’s approval for medical use in 1995, safety information and other data on docetaxel have been reported. One of the common toxicities of docetaxel treatment is neutropenia [6, 7], which can have a significant influence on the treatment of cancer and condition of the patient. In those who experience this side effect, careful observation and prophylactic treatment with granulocyte colony-stimulating factor (G-CSF) are recommended [8, 9]. Additionally, patients with risk factors such as advanced age, infectious diseases, recent surgical history, renal or hepatic impairment, and drug interactions may experience worsening of their neutropenia [8]. More safety and toxicity data must be collected in addition to the information published to date to ensure the effective and safe treatment of breast cancer.

However, in the case of anticancer drugs, clinical trials on safety and toxicity are limited due to the high risks of adverse events and ethical issues, and there are few studies that address drug interactions. Cladribine, natalizumab, and upadacitinib are the common drugs contraindicated for coadministration with docetaxel [10], and caution is required when some drugs, such as cytochrome P450 3A4 inhibitors and platinum derivative, are coadministered [1, 10, 11]. When docetaxel and the abovementioned drugs are coadministered, treatment limitations and toxicities should be determined based on clinical observations.

This study was undertaken to evaluate drug–drug interactions (DDIs) when drugs are coadministered with docetaxel to patients with breast cancer using a nationwide claims database and the risk of neutropenia under docetaxel administration or the coadministration of docetaxel and a predefined drug.

## Methods

### Data source

Nested case–control study data were obtained from the Health Insurance Review and Assessment Service (HIRA) database, which is a repository of claims data used to reimburse healthcare providers in the Korean National Health Insurance (NHI) system. The HIRA database includes encrypted patient information including identification numbers, ages, genders, types of insurance, procedures, diagnosis, prescriptions, medical institution identifiers, and records and whether patients were treated as inpatients or outpatients. The collected prescription information included drug codes, days of supply, doses, and routes of administration. Diagnoses were coded according to the Korean Standard Classification of Diseases (Seventh Revision (KCD-7)). We used HIRA sample databases for 2017, 2018, and 2019 in this study, which included 3% of all NHI beneficiaries. The study protocol was reviewed by the Institutional Review Board of Yeungnam University (YU202010003), and the requirement for informed consent was waived because secondary data were used. Data from the NHI exclude information that enables the identification of individuals.

### Study groups and outcome measures

Female breast cancer (KCD-7 code: C50) prescribed with docetaxel after their diagnosis of breast cancer and included in the HIRA patients sample database (2017–2019). Patients who met the following criteria were excluded: 1) a prescription of docetaxel or predefined interacting drugs before their first diagnosis of breast cancer, 2) a hematologic malignancy, and 3) a prescription of G-CSF before the prescription of docetaxel.

Interacting drugs were defined based on the approval information obtained from the Korean Ministry of Food and Drug Safety and the Lexicomp electronic database (S1 Table). Patients who had been prescribed G-CSF during the continuous administration of docetaxel were assigned to the case group (patients with G-CSF), while those who had not been prescribed G-CSF were assigned to the control group (patients without G-CSF). Continuous docetaxel administration was defined as the next administration of docetaxel identified within 28 days after the previous administration. The risk of neutropenia was defined as receipt of G-CSF prescriptions (S1 Table).

### Covariates and statistical analysis

Age, types of insurance, the period from first diagnosis of breast cancer to first administration of docetaxel, surgery for breast cancer, average dose of docetaxel administered per cycle, and Charlson Comorbidity Index (CCI) were evaluated [12, 13]. Age was assessed at the time of the initial diagnosis of breast cancer, and following the diagnosis, surgery for breast cancer was checked before and after administration of docetaxel. Insurance types were decided at the first prescription of docetaxel. The time from the initial diagnosis of breast cancer to the first prescription of docetaxel was also evaluated, and the average dose of docetaxel administered per cycle was calculated during the continuous prescription of docetaxel. The CCI was applied to the adjustment using the comorbidity diagnosis code prior to the initial diagnosis of breast cancer (S2 Table).

A nested case–control design was applied. In the nested case–control study, patients in the case group who had developed a disease during a specific time period were identified, and each patient was matched with other patients in the control group who had not developed the disease at the same period [14]. The propensity score matching (PSM) method was used to balance covariates in the case and control groups. PSM in the nested case–control study represents a conditional probability that patients will be assigned to the case group (with the prescription of G-CSF) under given covariates, including age, insurance types, the period from initial diagnosis of breast cancer to the first administration of docetaxel, surgery for breast cancer, the average dose of docetaxel administered per cycle, and CCI. Propensity scores (PSs) were calculated using a logistic regression model, in which the dependent variable was whether to assign the case group or the control group and the independent variables were the covariates mentioned above. The case and control groups were balanced for these covariates by 1:1 matching the PSs of individual patients by stratifying the administration of docetaxel into two strata (one cycle and more than two cycles). We evaluated standardized differences to determine a balance between groups before and after the PSM method; an absolute value for the standardized difference of <10% was a relatively small imbalance [15].

Odds ratios (ORs) for the association between receipt of docetaxel or docetaxel and a predefined drug and outcome (G-CSF prescriptions) were calculated using logistic regression models applied to the PSM method. In addition, along with the cycles for the administration of docetaxel used as a stratification variable in the PSM process, covariates were adjusted for the models, such as age, type of insurance, surgery for breast cancer type, the length of period from the initial diagnosis of breast cancer to the first prescription of docetaxel, average docetaxel dose per administration cycle, and CCI [16, 17].

Means, standard deviations, and percentiles were used as descriptive statistics. The statistical analysis was performed using an SAS version 9.4 (SAS Institute, Inc., Cary, NC, USA), and statistical significance was accepted for p-values <0.05.

For the validity assessment of the adjusted logistic regression model, we verified the linearity assumption for logit using the Box–Tidwell approach and determine whether there are any significant outliers with a Cook’s distance of greater than (4/the number of PS-matched patients) and absolute standardized residuals greater than 3. Additionally, the internal validation evaluation of the adjusted model was performed by yielding the value of AUC called an area under the ROC curve through 5-fold cross-validation [18–20]. This analysis was performed using an R software version 4.2.3.

## Results

### Population characteristics

**Fig 1:**
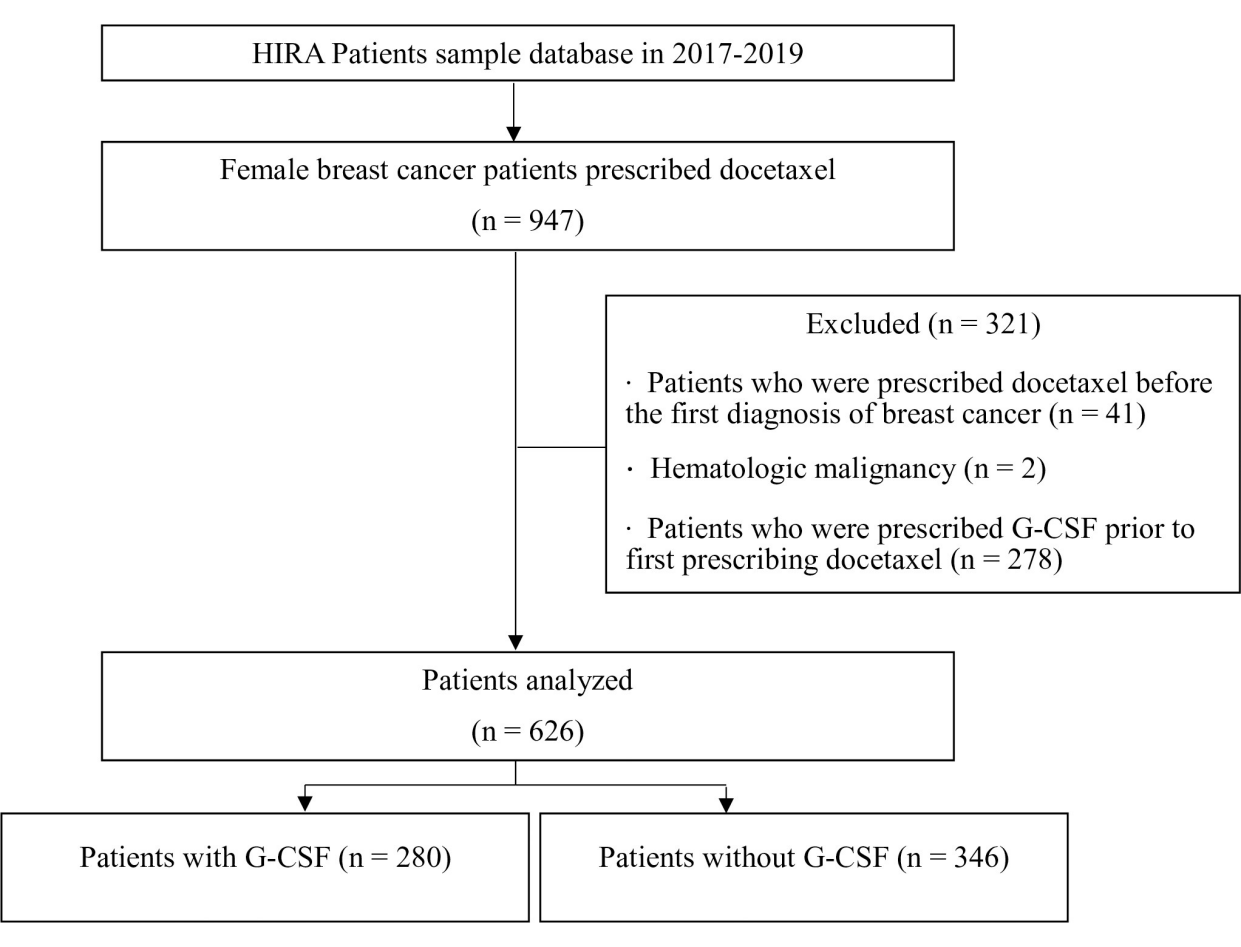
Flow chart of the patient selection process.

947 female patients with breast cancer prescribed with docetaxel were identified in the HIRA database (2017–2019). Excluded in this study were patients who were prescribed with docetaxel or interacting drugs before the initial diagnosis of breast cancer, patients with hematologic malignancy, or patients who were prescribed with G-CSF before the first docetaxel prescription. Of the 626 eligible patients, 280 of them had a record of G-CSF prescription (the case group), and the remaining 346 had no record of G-CSF prescription (the control group) (Fig 1). Patient baseline characteristics are presented in Table 1. When standardized differences of >0.1 were considered significant, age, types of insurance, surgery for breast cancer after the administration of docetaxel, the period from the first diagnosis of breast cancer to the first administration of docetaxel, and average dose of docetaxel per administration cycle differed between the two groups. Because of the PSM according to the stratified cycles (one cycle or more than two cycles) of administered docetaxel, 238 patients in the case group who were prescribed with G-CSF due to neutropenia at the administration of docetaxel were matched with 238 patients in the control group without G-CSF prescription. Differences between baseline characteristics were not significant in the two groups as all absolute values of standardized differences were less than 0.1 when 238 patients in the two groups were matched (Table 2). In other words, all covariates were balanced in both the case and control groups.

**Table 1:** Patient baseline characteristics before applying the propensity score matching.

Characteristics		Overall (N = 626)		Case (N = 280)		Control (N = 346)		Standardized difference (STD)
		N	(%)	N	(%)	N	(%)	
Age	50 less	283	(45.21%)	116	(41.43%)	167	(48.27%)	0.1378
	50 more than	343	(54.79%)	164	(58.57%)	179	(51.73%)	
Insurance	Health insurance	603	(96.33%)	276	(98.57%)	327	(94.51%)	0.2237
	Medical aid	23	(3.67%)	4	(1.43%)	19	(5.49%)	
Breast cancer surgery	Before the administration of docetaxel	276	(44.09%)	131	(46.79%)	145	(41.91%)	0.0983
	After the administration of docetaxel	104	(16.61%)	30	(10.71%)	74	(21.39%)	−0.2939
CCI (mean±SD)		0.42±1.14		0.46±1.08		0.40±1.18		0.0572
The period [days] from the first diagnosis of breast cancer to the first administration of docetaxel (mean±SD)		54.26±59.89		50.78±56.68		57.08±62.30		−0.1058
The average dose [mg/cycle] of docetaxel administered per cycle (mean±SD)		126.40±19.43		127.51±20.38		125.51±18.61		0.1023

**Table 2:** Patient baseline characteristics after applying a propensity score matching.

Characteristics		Overall (N = 476)		Case (N = 238)		Control (N = 238)		Standardized difference (STD)
		N	(%)	N	(%)	N	(%)	
Age	50 less	202	(42.44%)	104	(43.70%)	98	(41.18%)	−0.0510
	50 more than	274	(57.56%)	134	(56.30%)	140	(58.82%)	
Insurance	Health insurance	468	(98.32%)	234	(98.32%)	234	(98.32%)	0.0000
	Medical aid	8	(1.68%)	4	(1.68%)	4	(1.68%)	
Breast cancer surgery	Before the administration of docetaxel	231	(48.53%)	110	(46.22%)	121	(50.84%)	−0.0923
	After the administration of docetaxel	53	(11.13%)	28	(11.76%)	25	(10.50%)	0.0401
CCI (mean±SD)		0.45±1.17		0.45±1.07		0.45±1.27		0.0072
The period [days] from the first diagnosis of breast cancer to the first administration of docetaxel (mean±SD)		52.87±56.79		51.12±58.55		54.61±55.04		−0.0614
The average dose [mg/cycle] of docetaxel administered per cycle (mean±SD)		126.02±18.46		126.79±20.50		125.26±16.18		0.0830

### Case–control study of drug–drug interactions

Predefined drugs were coadministered with docetaxel 7 days before and after the administration of docetaxel in 71 (11.3%) patients. Although these combinations are not contraindicated, they should be used with caution. The most frequent combination was docetaxel plus carboplatin in both groups. Only 3 patients (0.5%) received DDI medications while receiving docetaxel therapy when anticancer medications were excluded from interactions with other drugs (Table 3).

**Table 3:** Number of patients who coadministered predefined drugs.

	Case (N = 32)	Control (N = 39)
Carboplatin	32	33
Cisplatin	-	2
Clarithromycin	-	4

Table 4 shows the ORs for associations between G-CSF prescriptions and docetaxel/coadministered drug interactions. Adjusted ORs calculated using the multivariate logistic regression model with stratified cycles (one cycle, more than two cycles) of administered docetaxel and covariates applied to the PSM method showed that the odds of G-CSF prescription were not significantly different in the case and control groups (adjusted OR, 2.010; 95% confidence interval [CI], 0.906, 4.459), that is, that docetaxel and predefined drug interactions were not associated with G-CSF prescription. We also evaluated the chi-square test for logistic regression models [16]. As a result, the data for both groups were not statistically significant, which indicates a homogeneous probability for the risk of G-CSF prescription.

**Table 4:** Associations between G-CSF prescription docetaxel and predefined drugs.

Drug exposure	With G-CSF [Case]	Without G-CSF [Control]	Crude OR (95% CI)	Adjusted OR* (95% CI)
*Before PSM*				
Docetaxel alone	248	307	1.00 (reference)	1.00 (reference)
Coadministration	32	39	1.016 (0.618, 1.669)	1.644 (0.883, 3.060)
*After PSM*				
Docetaxel alone	209	218	1.00 (reference)	1.00 (reference)
Coadministration	29	20	1.512 (0.830, 2.757)	2.010 (0.906, 4.459)

*Age, types of insurance, surgery for breast cancer, CCI, the period [days] from the first diagnosis of breast cancer to the first administration of docetaxel, average dose [mg/cycle] of docetaxel administered per cycle, and stratified cycles (one cycle, more than two cycles) of administered docetaxel were used for adjustment.

Furthermore, the assumption of linearity between continuous covariates (the period from the first diagnosis of breast cancer to the first administration of docetaxel, average dose of docetaxel administered per cycle, CCI) and the logit transformation of binary outcome in the adjusted logistic regression model was evaluated using the Box–Tidwell test. In order to apply this approach, we added an interaction term between each continuous covariate and its logarithmic transformation to the model, and if the added interaction term for each covariate is statistically insignificant, associations between the corresponding covariate and the logit of binary outcome will prove to be linear [18].

We showed the regression coefficient estimates (95% CI), p-value, and Bayes factor (BF) for the interaction terms of three continuous covariates. First, for CCI, the estimate was −0.459 (95% CI: −0.920, 0.002), the p-value was 0.051, and BF was 0.307. Although its p-value was obtained close to 0.05, its BF was lower than 3 to 5, which is the value of BF corresponding to the p-value of 0.05, so the interaction term for CCI was statistically insignificant [21]. The other interaction terms for two continuous covariates (the period from the first diagnosis of breast cancer to the first administration of docetaxel: the regression coefficient estimate was 0.001 (95% CI: −0.005, 0.007), the p-value was 0.758, and BF was 0.048; an average dose of docetaxel administered per cycle: the regression coefficient estimate was 0.065 (95% CI: −0.012, 0.141), the p-value was 0.099, and BF was 0.179) were also statistically insignificant. In conclusion, three continuous covariates had linear relationships with the logit of the binary outcome in the adjusted logistic regression model.

As further validity assessments for the adjusted logistic regression model, we demonstrated the absence of influential outliers from the 476 PS-matched patients (although 31 of 476 patients with the Cook’s distance greater than 0.0084 (= 4/476) were influential to the model, there were no PS-matched patients with absolute standardized residuals greater than 3) [19]. Finally, we drew the ROC curve and then yielded the value of AUC to show the predictive ability for G-CSF prescription from the adjusted outcome model (AUC: 0.856) (S1 Fig). We also performed 5-fold cross-validation to evaluate the internal validity of this model and obtained an average of the 5 AUC values (= 0.8385) [20].

## Discussion

We identified docetaxel DDIs using real-world claims data. In addition, the risks of neutropenia (defined as receipt of a prescription of G-CSF) for docetaxel alone and docetaxel combined with a predefined drug were compared to assess the association between drug interactions and adverse events.

In this study, 11% of the patients with breast cancer who received docetaxel concurrently used clinically significant interacting medications. When we excluded anticancer medications from interacting medications, the prevalence of DDIs was only 0.5%. To the best of our knowledge, this study is the first to evaluate the prevalence of docetaxel drug–drug interactions using a nationwide database. Although direct comparisons are not possible due to the use of different study designs, anticancer agents, and databases, the two reported overall prevalences of DDIs for anticancer medications were 32.4% or 26.4%, and when limited to significant drug–drug interactions, the prevalences for anticancer medications were 28.4% or 9.7% [22, 23].

Coadministration with carboplatin was the most frequent combination observed in this study, which results in a pharmacodynamic drug interaction. Docetaxel and carboplatin are often administered in combination because this therapy is recommended for patients with breast cancer [2]. Therefore, when this treatment is administered, the prophylactic use of G-CSF should be taken into account, and other risk factors of neutropenia should be assessed. Coadministration with clarithromycin or itraconazole can also induce a pharmacokinetic interaction [10]. Dronedarone is a medication that may induce a pharmacokinetic interaction in addition to cytochrome P450 3A4 inhibitors, although this has not been observed in the present study [10].

Patel *et al*. reported that the incidence of febrile neutropenia among patients with breast cancer who were administered with docetaxel plus cyclophosphamide was 9.4%, which is consistent with our findings [24]. Unfortunately, few studies have reported drug interactions for anticancer drugs. According to a retrospective cohort study that investigated the potential interaction between vinorelbine and clarithromycin, the estimated incidence of grade 4 neutropenia in patients receiving clarithromycin was higher than that in patients not receiving clarithromycin (31.6% and 2.5%, p = 0.0033) [25]. Additionally, it was reported that vinorelbine dose and female sex were significantly correlated with severe neutropenia. However, we failed to identify a significant difference in neutropenia risk. This difference might be explained by the high distribution rate of electronic medical records and clinical decision support systems for prescribing anticancer medications, which makes preemptive therapy modifications possible based on considerations of DDIs and other risk factors for adverse drug events. Our findings indicate no additional risk of neutropenia in patients with breast cancer who were prescribed with docetaxel and other potential interacting drugs. However, given the impact of DDIs, we suggest exercising caution when interpreting our results and recommend further studies be undertaken.

Studies on the safety of docetaxel using real-world data are valuable because they provide an alternative that is not encumbered by ethical or financial considerations and avoid the drawbacks of clinical trials carried out in controlled environments. Nevertheless, our study has several limitations. First, despite the benefits of using claims data, it can be challenging to determine if a drug was actually administered or when it was administered. Second, neutropenia was defined as an adverse effect due to DDI, but the confirmation of the relationship between DDI and the occurrence of the actual adverse drug event is unclear. Third, we could not evaluate factors associated with patient status, such as body weight and ECOG performance status, or anticancer medication doses, because these were not recorded in the claims database. Finally, the sample database excluded patients who were diagnosed with neutropenia. A history of neutropenia is a significant risk factor and could be likely to have influenced observed outcomes; therefore, exclusion is considered reasonable.

## Conclusions

This study indicates that coadministration of docetaxel and a predefined interacting drug is not associated with prescription of G-CSF. However, this does not support the idea that drugs that cause potential neutropenia with docetaxel might be used safely. Moreover, this study shows that investigations based on real-world data make it possible to monitor and evaluate cancer treatment safety, thus improving patient care.

## Supporting information

S1 Table Medication ingredient codes of predefined drugs and G-CSF. (DOCX)

S2 Table Charlson comorbidity indices (CCIs). (DOCX)

S1 Fig ROC curve for the PS−matched logistic regression model. (PDF)
