# Supplementary material for: Drug–Drug interactions of docetaxel in patients with breast cancer based on insurance claims data
Source: PLoS One. 2023 Jun 16;18(6):e0287382. doi: 10.1371/journal.pone.0287382 (PMC10275435; doi:10.1371/journal.pone.0287382)
Supplement: S1 Table — (DOCX) [file pone.0287382.s001.docx]

S1 Table. Medication Ingredient Codes of pre-defined drugs and G-CSF

| Drug | Code |
| --- | --- |
| *Interacting drugs* | |
| Carboplatin | 123730BIJ, 123731BIJ, 123732BIJ, 123733BIJ, 123734BIJ, 123735BIJ |
| Cisplatin | 134530BIJ, 134533BIJ, 134534BIJ |
| Clarithromycin | 134935ASY, 134937ASY, 134901ATB, 134904ATB, 134904ATR, 134902BIJ |
| Itraconazole | 179131ALQ, 179101ACH, 179101ATB, 179104ATB |
| *G-CSFs* | |
| Filgrastim | 158930BIJ, 158931BIJ, 158932BIJ, 158933BIJ, 158934BIJ, 158935BIJ, 158936BIJ |
| Lenograstim | 181801BIJ, 181802BIJ, 181803BIJ |
| Pegfilgrastim | 618830BIJ |
| Lipegfilgrastim | 684901BIJ |
