# Supplementary material for: Drug–Drug interactions of docetaxel in patients with breast cancer based on insurance claims data
Source: PLoS One. 2023 Jun 16;18(6):e0287382. doi: 10.1371/journal.pone.0287382 (PMC10275435; doi:10.1371/journal.pone.0287382)
Supplement: S2 Table — (DOCX) [file pone.0287382.s002.docx]

S2 Table. Charlson comorbidity indices (CCIs)

| Code | Disease | Weights | Diagnose code |
| --- | --- | --- | --- |
| 1 | Acute myocardial infarction | 1 | I21, I22, I252 |
| 2 | Congestive heart failure | 1 | I50 |
| 3 | Peripheral vascular disease | 1 | I71, R02, 1190, I739, Z958, Z959 |
| 4 | Cerebral vascular accident | 1 | 160, I61, I62, I63, I65, I66, G64, I64, I69  G450, G451, G452, G458, G459, I670, I671, I672, I674, I675, I676, I677, I678, I679, I681, I682, I688 |
| 5 | Dementia | 1 | F00, F01, F02, F051 |
| 6 | Pulmonary disease | 1 | J40, J41, J42, J43, J44, J45, J46, J47, J67, J60, J61, J62, J63, J66, J64, J65 |
| 7 | Connective tissue disorder | 1 | M32, M34, M332, M053, M058, M059, M060, M063, M050, M052, M051, M353 |
| 8 | Peptic ulcer | 1 | K25, K26, K27, K28 |
| 9 | Liver disease | 1 | K702, K703, K73, K717, K740, K742, K746, K743, K744, K745 |
| 10 | Diabetes | 1 | E109, E119, E139, E149, E101, E111, E131, E141, E105, E115, E135, E145 |
| 11 | Diabetes complications | 2 | E102, E112, E132, E142, E103, E113, E133, E143, E104, E114, E134, E144 |
| 12 | Paraplegia | 2 | G81, G041, G820, G821, G822 |
| 13 | Renal disease | 2 | N03, N01, N18, N19, N25, N052, N053, N054, N055, N056, N072, N073, N074 |
| 14 | Cancer | 2 | C0, C1, C2, C3, C5, C6  C40, C41 |
| 15 | Severe liver disease | 3 | K729, K766, K767, K721 |
| 16 | Metastatic cancer | 6 | C77, C78, C79, C80 |
| 17 | HIV | 6 | B20, B21, B22, B23, B24 |

CCI score was calculated by (CCI_1 + CCI_2 + CCI_3 + CCI_4 + CCI_5 + CCI_6 + CCI_7 + CCI_8 + CCI_9 + CCI_10)*1 + (CCI_11 + CCI_12 + CCI_13 + CCI_14)*2 + CCI_15*3 + (CCI_16 + CCI_17)*6
