# Supplementary figures and images for: Drug–Drug interactions of docetaxel in patients with breast cancer based on insurance claims data
Source: PLoS One. 2023 Jun 16;18(6):e0287382. doi: 10.1371/journal.pone.0287382 (PMC10275435; doi:10.1371/journal.pone.0287382)

ROC\_Curve for the PS-Matched logistic regression model

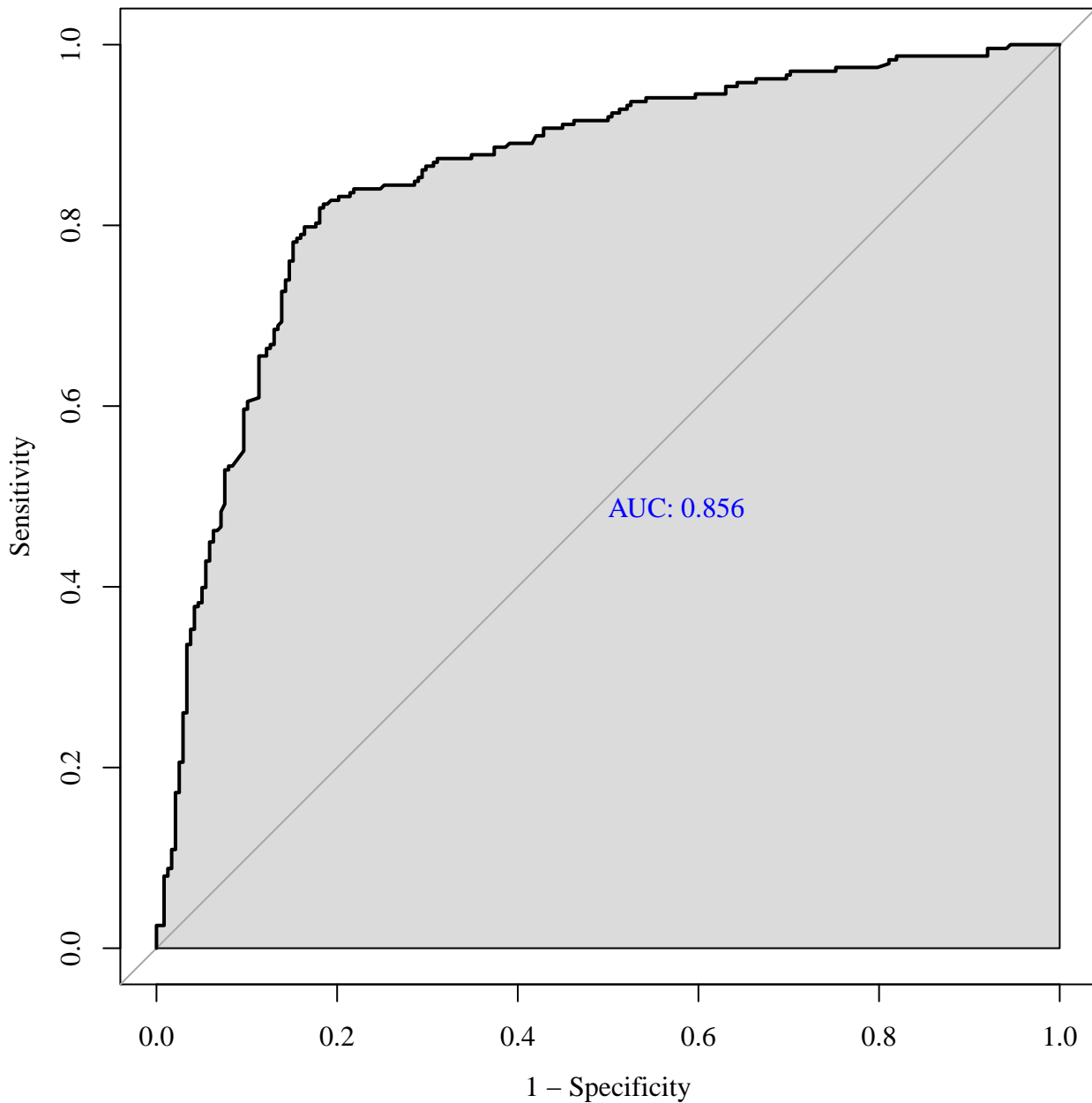

Supplement: S1 Fig — (PDF) [file pone.0287382.s003.pdf]
